# Safety in the shallows: nearshore coastal habitats can provide physical and thermal features that optimize escape performance in newborn blacktip reef sharks (*Carcharhinus melanopterus*)

**DOI:** 10.1093/conphys/coaf045

**Published:** 2025-08-04

**Authors:** José E Trujillo, Ian A Bouyoucos, Ornella C Weideli, Elena M C Milanesi, Shamil F Debaere, William J Rayment, Serge Planes, Paolo Domenici, Jodie L Rummer, Bridie J M Allan

**Affiliations:** Department of Marine Science, University of Otago, 362 Leith Street North, Dunedin 9016, New Zealand; Australian Research Council Centre of Excellence for Coral Reef Studies, College of Science and Engineering, James Cook University, 1 James Cook Dr, Townsville, QLD 4811, Australia; PSL Research University, EPHE-UPVD-CNRS, USR 3278 CRIOBE, 68 Avenue Paul Alduy Université de Perpignan, 66860 Perpignan, Cedex, France; PSL Research University, EPHE-UPVD-CNRS, USR 3278 CRIOBE, 68 Avenue Paul Alduy Université de Perpignan, 66860 Perpignan, Cedex, France; Institute of Laboratory Medicine (ILM), Private University in the Principality of Liechtenstein (UFL), Dorfstrasse 24, FL-9495 Triesen, Principality of Liechtenstein; Dr Risch Medical Laboratory, Wuhrstrasse 14, 9490 Vaduz, Liechtenstein; Department of Earth and Environmental Sciences (DISAT), University of Milano - Bicocca, Piazza della Scienza, 1, Milano 20126, Italy; ECOSPHERE, Department of Biology, University of Antwerp, Groenenborgerlaan 171, 2020 Antwerp, Belgium; Marine Biology, College of Science and Engineering, James Cook University, 1 James Cook Dr, Townsville, QLD 4811, Australia; Department of Marine Science, University of Otago, 362 Leith Street North, Dunedin 9016, New Zealand; PSL Research University, EPHE-UPVD-CNRS, USR 3278 CRIOBE, 68 Avenue Paul Alduy Université de Perpignan, 66860 Perpignan, Cedex, France; Laboratoire d’Excellence ‘CORAIL’, EPHE, PSL Research University, UPVD, CNRS, USR 3278 CRIOBE, BP 1013 Papetoai, 98729 Moorea, French Polynesia; CNR, Institute of Biophysics, Area di Ricerca San Cataldo, Via G. Moruzzi N°1, 56124 Pisa, Italy; Australian Research Council Centre of Excellence for Coral Reef Studies, College of Science and Engineering, James Cook University, 1 James Cook Dr, Townsville, QLD 4811, Australia; Marine Biology, College of Science and Engineering, James Cook University, 1 James Cook Dr, Townsville, QLD 4811, Australia; Department of Marine Science, University of Otago, 362 Leith Street North, Dunedin 9016, New Zealand

**Keywords:** Aerobic scope, antipredator behaviour, fast-start escape responses, oxygen uptake rate, predator–prey interactions, shark nursery areas

## Abstract

The prevailing shark nursery paradigm suggests that high survival in these habitats is primarily driven by reduced predator encounters: so-called pre-encounter risk. In this study, we propose an alternative or complementary mechanism: that some nurseries may lower post-encounter risk by providing environmental conditions that maximize escape performance. To test this hypothesis, we examined how temperature, depth and habitat complexity influence the escape performance of newborn blacktip reef sharks (*Carcharhinus melanopterus*) in Mo′orea, French Polynesia. In a controlled setting, we exposed 48 newborn sharks to four temperature treatments (25, 27, 29 and 31°C) and measured fast-start acceleration, turning rate and latency to respond to a stimulus. We also calculated aerobic scope at 27, 29 and 31°C, as greater aerobic scope is associated with faster recovery from burst swimming. Our results show that warmer temperatures improve escape performance, with 29% higher acceleration, 9% faster turning rates and 48% shorter reaction times at elevated temperatures. Furthermore, aerobic scope remained ≥80% of its maximum capacity between 27.5 and 30.8°C, suggesting that newborn sharks can sustain high metabolic performance within this thermal window. Field measurements at nursery habitats revealed that daily thermal fluctuations generally remained within this optimal aerobic scope range, meaning that newborns can maintain high escape performance for most of the day. Additionally, high-resolution mapping confirmed that previously reported home ranges were associated with shallow (median depth = 0.74 m), structurally complex reef flats dominated by coral substrate. The combination of reduced hydrodynamic drag in shallow water and increased manoeuvrability in complex habitats likely enhances predator evasion. However, extreme warming events that exceed critical thermal limits may trigger behavioural trade-offs that compromise escape performance and elevate predation risk. Our findings suggest that these nurseries provide habitat-specific advantages for predator evasion, reinforcing their critical role in the survival of newborn sharks.

## Introduction

Many shark species use nearshore coastal habitats as nursery areas, where juveniles find food and refuge as they grow (Gruber *et al*., 2001; Heupel and Simpfendorfer, 2002; Kinney and Simpfendorfer, 2009). These nurseries are typically characterized by the exclusion of large predators (Paterson and Whitfield, 2000; Beck *et al*., 2001; Baker and Sheaves, 2007). Specifically, it has been suggested that young sharks inhabit areas spatially segregated from adult conspecifics (Springer 1967), as intraspecific predation risk is notably high among sharks (Clarke, 1971; Branstetter, 1990; Guttridge *et al*., 2012). The prevailing shark nursery paradigm suggests that the reduced likelihood of encountering predators—referred to as pre-encounter risk (Lima and Dill, 1990; Hebblewhite *et al*., 2005)—is the primary driver behind the high survival rates observed in these habitats (Heupel and Hueter, 2002; Simpfendorfer *et al*., 2005; Heupel *et al*., 2007; Heupel and Simpfendorfer, 2011; Heupel *et al*., 2018). Consequently, minimizing pre-encounter risk has been a central tenet in the shark nursery hypothesis. However, this assumption may not hold true for all nurseries (see Heupel *et al*., 2007 for a review). We propose an alternative or complementary mechanism whereby sharks minimize post-encounter risk within nurseries that provide environmental conditions that allow for maximal escape performance, rather than solely reducing the likelihood of predator encounters.

Antipredator escape tactics like fast-start escape responses play an important role in reducing post-encounter risk (Domenici and Blake, 1997; Domenici and Hale, 2019). These rapid evasive manoeuvres are prevalent among fish, including small sharks, and can be effective in predator-rich environments (Domenici *et al*., 2004, Walker *et al*., 2005). Fast-start escape responses are vital to escape from a predator’s capture zone, once it strikes. Trujillo *et al*. (2022) showed that newborn blacktip reef sharks (*Carcharhinus melanopterus*), a nursery-bound species (Bouyoucos *et al*., 2022), display fast-start escape responses with latencies that are among the shortest observed when considering both teleosts and elasmobranchs. Nurseries for *C. melanopterus* are also foraging microhabitats for adult conspecifics (J.E. Trujillo, personal observations; Papastamatiou *et al*., 2009; Papastamatiou *et al*., 2015,), and it is likely they are exposed to high pre-encounter risk. Thus, fast-start escape responses may be crucial for *C. melanopterus* newborns to mitigate post-encounter predation risks. Indeed, prey with substantial escape abilities have lower post-encounter risk and can inhabit areas of high predator densities, contrary to prey with limited escape abilities that rather avoid predator areas (Wirsing *et al.*, 2010). Maximal escape performance is especially critical for juvenile survival, as they must achieve a significant proportion of their maximum locomotor capacity to survive predator attacks (Husak, 2006; e.g. see performance space, Bennett, 1989). In particular, for naïve individuals with limited experience of predator encounters, achieving maximal escape performance is even more critical for survival, as their lack of experience may hinder their ability to appropriately assess risks or optimize escape strategies (Ramasamy *et al*., 2015; Domenici, 2010).

Understanding how environmental conditions influence escape performance in nursery-bound sharks is crucial for identifying the environmental drivers of post-encounter survival and refining our understanding of nursery habitat function. Escape behaviour is widely recognized as a key factor shaping prey spatial responses to predation (e.g. see Dellinger *et al*., 2019). In marine environments, Indo-Pacific bottlenose dolphins (*Tursiops aduncus*), dugongs (*Dugong dugon*) and green turtles (*Chelonia mydas*) move into the shallow waters of Shark Bay, Australia—despite the presence of predators like tiger sharks (*Galeocerdo cuvier*)—because the shallows are thought to improve the effectiveness of their escape tactics (Heithaus *et al*., 2009). In contrast, pied cormorants (*Phalacrocorax varius*) avoid the shallow areas frequented by tiger sharks because they do not gain any advantage escaping in shallow water (Heithaus *et al*., 2009). Similarly, certain environmental conditions within shark nurseries may enhance escape performance in young sharks, potentially giving them a survival advantage against predators compared to other habitats. However, whether this is a defining characteristic of all shark nurseries remains an open question.

Environmental factors such as temperature, depth and habitat structure significantly influence escape performance. Temperature affects overall escape performance in ectotherms, such as fish (Domenici *et al*., 2019). Warmer temperatures increase fast-start turning rate, maximum acceleration and speed (Wardle, 1975; Webb, 1978b; Randall and Brauner, 1991; Wakeling, 2005). Warmer temperatures also speed up the brainstem escape network in fish (Webb, 1978a; Preuss and Faber, 2003), accelerating their escape reaction times (i.e. reduced escape latency in damselfish *Pomacentrus wardi*  Allan *et al*., 2015 and goldfish *Carassius auratus*, Szabo *et al*., 2008), and significantly increasing their survival rates (McCormick *et al.*, 2018). Furthermore, temperature can indirectly influence escape performance by affecting a fish’s scope for activity, measured as the difference between maximum and standard (basal) metabolic rates (i.e. Aerobic Scope; AS). The aerobic scope–drive hypothesis is based on the premise that anaerobically powered fast-start escape responses necessitate a metabolic recovery phase that is fuelled aerobically (Killen *et al*., 2015). Fish use a portion of their AS to metabolize the waste products accumulated during anaerobic exercise (Gaesser and Brooks, 1984; Richards *et al*., 2002; Richards *et al*., 2003). Consequently, fish with larger AS recover faster from exercise (Marras *et al.*, 2010). Because AS peaks at an optimal temperature in ectotherms (Fry, 1947; Pörtner, 2002; Gannon *et al*., 2014; Pörtner *et al*., 2017; Rezende and Bozinovic, 2019), habitats near the AS optimum can promote short recovery from exercise such as that due to escaping from predator attacks.

Small prey often outmanoeuvre larger predators due to their superior turning performance (Webb, 1976), a trait that is especially advantageous in structurally complex habitats and shallow waters (Webb, 1976; Domenici and Blake, 1997; Blake, 2009). The structural complexity of habitats can reduce the swimming ability of large predators, thereby impeding their foraging efficiency (Manatunge *et al*., 2000). Similarly, shallow water increases drag on predators during attack strikes as they approach the surface (Hertel, 1966; Webb *et al*., 1991). This drag is determined by the submergence depth index (*z*/*B*), where *z* is the depth from the surface to the centre line of the object, and *B* is the span of the object (Blake, 2009). Drag becomes negligible only when *z*/*B* ≥ 3 (Blake, 2009), meaning that if fish initiate fast-start escape responses near the surface (i.e. *z*/*B* < 3), they experience a decline in performance (Webb *et al*., 1991). This drag effect is stronger in larger individuals, such as predators, giving small prey an advantage in shallow environments due to their large submergence index. This raises the question of whether shark nurseries provide the structural complexity and water depth necessary to facilitate maximal escape performance.

In Mo′orea, French Polynesia, *C. melanopterus* newborns use nearshore habitats as nurseries (Bouyoucos *et al*., 2022). The newborns display limited spatial distribution characterized by small home ranges of ~0.02–0.14 km^2^ (Bouyoucos *et al*., 2020b). Tracking data shows that their habitat use is exclusively limited to the shallow flats near the coast (Bouyoucos *et al*., 2020b). This site fidelity to habitats that concurrently serve as foraging microhabitats for adult conspecifics (Papastamatiou *et al*., 2009; Papastamatiou *et al*., 2015) suggests that pre-encounter predation risk is high within the proposed nurseries, due to the high risk of intraspecific predation among sharks (see above). Alternatively, *C. melanopterus* newborns may have low post-encounter predation risk because they can attain a high proportion of their maximal escape capacity within the nurseries. We hypothesized that the environmental conditions (i.e. temperature, depth and topographic configuration) within Mo′orea’s nurseries promote maximal escape performance by reducing latency, improving agility and decreasing predator efficiency. We also hypothesized that water temperature within these nurseries approximates the thermal optimum for AS, aligning with the average body temperature of neonates observed in the wild (29.6°C), as suggested by Bouyoucos *et al*. (2020a). By integrating laboratory and field data, we examined the relationships between habitat features and newborn blacktip reef shark performance to better understand their survival strategies.

## Materials and Methods

### Ethical note

All procedures were approved under James Cook University Animal Ethics Committee protocols A2394 and A2769. Shark research in French Polynesia was approved under *Arrêté* N° 11 491 and N° 5129 issued by the *Ministrère de la Promotion des Langues, de la Culture, de la Communication et de l’Environnement* of the French Government on 16 October 2019 and 22 June 2016, respectively. All experiments were conducted in the *Centre de Recherches Insulaires et Observatoire de l’Environnement* (CRIOBE) facilities. All newborn sharks used in our laboratory experiments were released back to their original site of capture, unless otherwise stated. To allow for recovery, sharks were fed immediately after procedures and left undisturbed for a period of 2–3 days prior to release (see Trujillo *et al*., 2022). Adult sharks were released immediately after *in situ* procedures.

### Study site and species

The island of Mo′orea is in the Society archipelago in French Polynesia (17°32′0″S, 149°50′0″W), in the South Pacific. Mo′orea is a volcanic island with a 60-km-long coastline encircled by a barrier reef (Madi Moussa *et al*., 2019). The coral reef–lagoon system of Mo′orea covers an area of 50.63 km^2^, encompassing the region from the outer reef to the shoreline (Kennedy *et al*., 2021; Allen Coral Atlas, 2022). For this study, we collected *C. melanopterus* newborns (*n* = 48) during two parturition seasons (November 2019–January 2020 and November 2021–January 2022; hereon seasons), from six sites around Mo′orea (see details in Fig. S1) using monofilament gillnets (50 × 1.5 m with 5-cm mesh size) at dusk (17 h00–20 h00). Following Debaere *et al*. (2023), newborns were identified by the presence of an open (unhealed) umbilical wound, which is used to estimate neonatal age classes, and were therefore considered to be <1 month old.

Adult *C. melanopterus* were caught with hook and line from a boat in the lagoon of Mo′orea to photograph their caudal fins to estimate submergence depth index (see ‘Submergence Depth Index’). Sharks were restrained on their backs on the side of the boat to induce tonic immobility. The head of the shark remained underwater to allow for buccal pumping. The caudal fin was lifted above the water surface to be photographed against the side of the boat using a scale. The entire procedure, which included additional body measurements and tissue sampling for parallel studies, lasted 9.4 ± 0.7 min from capture to release, thus minimizing post-release stress responses (Hassanein, 2011; Harding *et al*., 2022).

### Animal husbandry

Newborn sharks were transported by vehicle to the CRIOBE research station in 200-l insulated coolers filled with aerated seawater (Bouyoucos *et al*., 2018). Total confinement time before arriving at the CRIOBE facilities was <90 min, and no injuries associated with this capture/transport method were observed. The morning following capture, sharks were marked with passive integrated transponder (PIT) tags (Biomark; www.biomark.com) inserted below the first dorsal fin for individual identification, measured and weighed. Sharks were 0.58 ± 0.1 m in total length (mean ± SE, range: 0.51–0.70 m) and 0.93 ± 0.03 kg in mass (range: 0.56–1.56 kg). Sharks (*n* = 48) were maintained in 1250-l circular tanks in groups of three with flow-through filtered seawater and aeration and covered with 60% shade cloth. The open-sided facilities provided a natural photo period. After 1 day in the laboratory, individuals were fed every 48 h with fresh tuna at 3–5% of their body mass.

### Habitat features

Active acoustic tracking data has shown that *C. melanopterus* newborns remain within the shallow flats around the coast of Mo′orea, using them as nurseries and avoiding deeper waters of the lagoon (Bouyoucos *et al*., 2020b). We characterized the topographic configuration, water temperature and depth within these nurseries using *in situ* and remotely sensed data. Habitat maps were then overlapped with home range data from Bouyoucos *et al*. (2020b).

#### Topographic configuration: geomorphic zones and benthic classes

We used the ‘Allen Coral Atlas’, a shallow-water coral reef map obtained from Planet Dove 3.7-m-resolution daily satellite imagery, to classify Mo′orea’s reef–lagoon system in geomorphic zones and benthic classes (Lyons *et al*., 2020; Allen Coral Atlas, 2022). Geomorphic zones were 1) reef slope, 2) sheltered slope, 3) reef crest, 4) outer reef flat, 5) inner reef flat, 6) terrestrial reef flat, 7) back reef slope, 8) deep lagoon, 9) shallow lagoon, 10) plateau, 11) patch reef and 12) small reef; and benthic classes were 1) coral/algae, 2) sand, 3) rubble, 4) rock, 5) seagrass and 6) microalgal mat (Kennedy *et al*., 2021). A detailed description of each classification can be found on the atlas’s website (www.allencoralatlas.org). We calculated the proportion of each benthic class within each of the geomorphic zones to determine topographic configuration.

#### Water temperature

Water temperature was measured using two temperature data loggers (*EnvTemperature*, ElectricBlue) placed concurrently (15–20 m apart) in four sites around Mo′orea, namely Papetoai, Maharepa, Haapiti and Vaiare (Fig. S1). These sites were selected as they were evenly distributed around the coast of Mo′orea. The loggers were synchronized to record temperature exactly at the top of each hour, with a 60-min interval (resolution: 0.1 or 0.5°C), from 21 November 2019 to 23 March 2023. Depths of loggers ranged between 0.30 and 1.5 m, avoiding air exposure in all cases. The loggers have a thermal inertia (time to reach 90% of a sudden change in temperature) of 5 min and 15 s, an average precision of ≤0.1°C, and an average accuracy of ≤0.2°C (ElectricBlue). These parameters provided reliable estimates of water temperature (e.g. see Staines *et al*., 2022).

The hourly temperatures recorded by the two data loggers were averaged for each site and for each of the 4 months when *C. melanopterus* newborns are observed within the flats (i.e. November, December, January and February). That is, each site and month had 4 years of hourly temperature records. We used these records to obtain a 24-h thermal cycle for each site and month. We fitted a locally estimated scatterplot smoothing (LOESS) using the *loess* function from the *stats* package in *R*. Smoothing parameters (degree and α) were selected using a cross-validation approach described by Trujillo *et al*. (2022). We derived the following parameters from the resulting fits: mean, minimum and maximum temperatures, time of minimum and maximum temperatures and temperature range. The thermal cycles were contrasted with the temperature for peak absolute aerobic scope (AAS) and the performance breadth (i.e. the range of T° where AAS ≥ 80%). We also calculated a thermal volatility index by measuring the standard deviation (SD) from the raw temperature records at each hour of the day for each month independently. Positive values indicate warming, whereas negative values indicate cooling. Therefore, the thermal volatility index indicated the risk that the thermal conditions at a specific time of the day would vary from the predicted temperature.

#### Bathymetry

A high-resolution bathymetric map was created in QGIS version 3.30.2 with 0.5-m resolution topo-bathymetric data from the 2015 airborne LiDAR survey that covered the entire island of Mo′orea. The survey was commissioned by the *Servic’ d’ l’Urbanisme de Polynésie française* (SAU) and managed by the *Service hydrographique et océanographique français* (Shom) (Shom-SAU-NSF, 2015). The resulting product, *Moorea 2015* *V. 20 171 015*, has an open data licence and is available at the Shom website (www.diffusion.shom.fr) where details on the sensors used and data resolution can be found. The tides in Mo′orea’s lagoon are remarkably small, ~0.2-m amplitude at spring tide (Hench *et al*., 2008), such that water circulation and exchange are minimal due to tidal fluctuation (Leichter *et al*., 2012). Finally, we calculated the median depth within each of the geomorphic zones found around Mo′orea in QGIS version 3.30.2 using the LiDAR-derived bathymetry maps. Further, bathymetry profiles were obtained from digital transects drawn perpendicular to the coast in the four study sites (Fig. S1).

### Laboratory experiments

#### Thermal exposure

Sharks were maintained under natural thermal conditions normally experienced by *C. melanopterus* newborns in the wild (range of body temperatures: 26.1–34.1°C, Bouyoucos *et al.*, 2020a). Specifically, water temperatures in the holding tanks had a day–night fluctuation between ~25°C and no more than 32°C. After 5 days under these natural conditions, we measured their escape performance and metabolic rates at four (25, 27, 29 and 31°C) and three (27, 29 and 31°C) temperature treatments, respectively. A total of 12 sharks were tested per temperature. To do so, on Day 6, once the water temperatures reached 2°C above or below the desired temperature, water temperature in the tank was stabilized using chillers (TK-1000/2000, TECO) and aquarium heaters (Tetra Aquarium HighPerformance Heater HT100). From this point on, we either cooled or heated the water at a rate of 0.5°C day^−1^ until the desired treatment temperature was reached on Day 9. That is, sharks experienced a total temperature change of 2°C over a 4-day period (Days 6–9 inclusive). During this period (Days 6–9), sharks no longer experienced day–night temperature changes. Sharks were fasted for 48 h (Days 8 and 9) and maintained at the treatment temperature for 24 h (Day 9) before trials on Day 10. We followed this procedure to ensure that body temperatures reached the desired testing temperature while preventing cold snap or heat shock effects.

#### Measurements of escape performance

After the 24-h thermal exposure, individual sharks were stimulated three consecutive times to induce an escape response following procedures described by Trujillo *et al*. (2022). Briefly, sharks were introduced to a test arena and allowed to acclimate for 2 h. The test arena consisted of a circular pool (3.4 m in diameter) with water depths between 16 and 19 cm to simulate their natural environment (see extended methods in supplementary material). We used a sudden mechano-acoustic stimulation consisting of a tapered steel weight dropped on to the surface of the water close to the shark’s head. Sharks were allowed to re-acclimate for 20 min between startles (Trujillo *et al*., 2022). Water temperature in the test arena matched the corresponding treatment temperature. Escape responses were video recorded from above at 240 frames per second and videos were post-processed to control for image distortion and parallax effects (see Trujillo *et al*., 2022 and extended methods in supplementary material). Immediately after the third escape response, sharks were individually chased for 3 min in the test arena and exposed to air for 1 min to simulate an exhaustive pursuit (e.g. exhaustive protocol, Clark *et al*., 2013; see next section). We only chased sharks from the 27, 29 and 31°C groups to estimate metabolic rates immediately after escape trails. Sharks from the 25°C group were returned to their holding tanks due to space and resource limitations to run additional experiments with this group.

Undistorted video sequences were analysed in ImageJ v2.0.0 (National Institute of Health) following procedures described by Trujillo *et al*. (2022). Fast-start escape responses consist of two contralateral body bends defined as Stage 1 (first body bend) and Stage 2 (second body bend) (Domenici and Blake, 1997). As such, escape responses were classified as single-bend (only Stage 1 present) and double-bend (Stage 1 and 2 present) responses (Domenici and Blake, 1997; Trujillo *et al*., 2022). Five performance variables were obtained for analyses:

1) Responsiveness: the proportion of escape responses induced out of the total number of stimulations.
2) Escape latency (in milliseconds): the delay in time between stimulus onset and escape onset.
3) Maximum Stage 1 turning rate (ω_S1_, degrees s^−1^): the maximum change in body angle with respect to time during Stage 1.
4) Maximum speed (*U*_MAX_, m s^−1^): the maximum speed of the centre of mass throughout the entire escape response (i.e. Stage 1 and 2).
5) Maximum acceleration ($\mathcal{a}$_MAX_, m s^−2^): the maximum acceleration throughout the entire escape response.

Please refer to Trujillo *et al*. (2022) for full details on lens distortion correction, tracking calibration and variable calculations. Maximal escape performance measured in the laboratory is a strong indicator of performance during escapes from predators in the lab (Walker *et al*., 2005) and in the wild (Irschick and Losos, 1998). The fastest escape latency, turning rate, speed and acceleration of the three responses was considered the estimate of maximal performance (Webb, 2004; Artacho *et al*., 2013). Notice that the lowest escape latency value is the fastest response as this is a measure of response time. We tested the effect of temperature on escape latency, ω_S1_, *a*_MAX_, *U*_MAX_, using linear regression with temperature as the continuous explanatory variable. Escape latency was log-transformed to meet model assumptions since raw latency showed heterogeneity of variances. We used the 5–20 ms latency range to a mechanical stimulus (Domenici and Hale, 2019) as a reference to interpret escape latency results.

#### Respirometry and oxygen uptake metrics

Immediately following the exhaustive protocol (see above; Clark *et al*., 2013), sharks were placed into individual respirometry chambers. Dissolved oxygen concentration (DO, in mg l^−1^) was measured every 2 s for 5 min (O_2_ measuring period) at 10-min intervals (flush period) for 24 h post-exercise yielding 14 400 DO readings and 96 oxygen decline slopes for each shark (4 slopes per hour). From the oxygen decline slopes we calculated a total of 96 oxygen uptake rate (*Ṁ*_O2_ in mg O_2_ h^−1^ kg^−1^) determinations per individual. Our respirometry protocol was similar to that used by Bouyoucos *et al*. (2020a) where comprehensive methodological details can be found (see extended methods in supplementary material).

From the post-exercise *Ṁ*_O2_ determinations we calculated the AAS as the difference between minimum *Ṁ*_O2_ (*Ṁ*_O2Min_) and maximum *Ṁ*_O2_ (*Ṁ*_O2Max_). *Ṁ*_O2Min_ was calculated as the mean of the lowest 10% *Ṁ*_O2_ values (Chabot *et al*., 2016) from the 24-h trial (i.e. 96 *Ṁ*_O2_ determinations). *Ṁ*_O2Max_ was calculated as the highest rate of oxygen uptake using a 30-s rolling regression, one sample unit at a time (i.e. overlapping), during the first hour post-exercise (i.e. from 4 linear declines in DO) with the function *auto_rate* from the R package *respR* (Harianto *et al*., 2019). This method scans the entire oxygen decline slope (four slopes) in search of the highest rate in oxygen decline accounting for the dynamic nature of *Ṁ*_O2_ in the absence of activity data (Zhang *et al*., 2019). We scaled *Ṁ*_O2Min_, *Ṁ*_O2Max_ and AAS to 1-kg of mass shark using the intraspecific metabolic scaling exponent of 0.89 such that all metrics were expressed in mg O_2_ h^−1^ kg^-0.89^ (Jerde *et al*., 2019).

We used a non-linear least squares (NLLS) regression approach to estimate aerobic thermal performance using the AAS following Padfield *et al*. (2021). First, the two most plausible functions to describe the relationship between temperature and performance were fitted to the data (i.e. Gaussian and Quadratic Angilletta, 2006). Since NLLS regression is sensitive to starting parameters, we ran 64 iterations with different starting values and selected the best fit for each function based on Akaike’s information criterion (AIC) scores with the *nls_multstart* function from the *nls.multstart* package (Padfield and Matheson, 2018). The best fit for each function was compared and the best function was selected based on AIC corrected for small sample sizes (AICc). We used diagnostic plots to check heteroskedasticity and autocorrelation in the residuals of the best model with the *nlsResiduals* function from the *nlstools* package (Baty *et al*., 2015). Finally, we extracted the following parameters from the best model using the *rTPC* package (Padfield *et al*., 2021): maximum AAS, temperature for peak AAS and performance breadth (using the 80% of the maximum AAS obtained from the fit).

Model uncertainty was estimated using a residual bootstrapping approach with the *Boot* function from the *car* package (Fox, 2007). From the bootstrapped distribution we calculated the bias-corrected and accelerated 95% confidence interval (bca 95% CI) for the model fit and the derived parameters using the same *R* function. A pairwise Holm–Sidak *post hoc* test (Holm, 1979) was used to compare mean AAS between temperatures.

**Figure 1:**
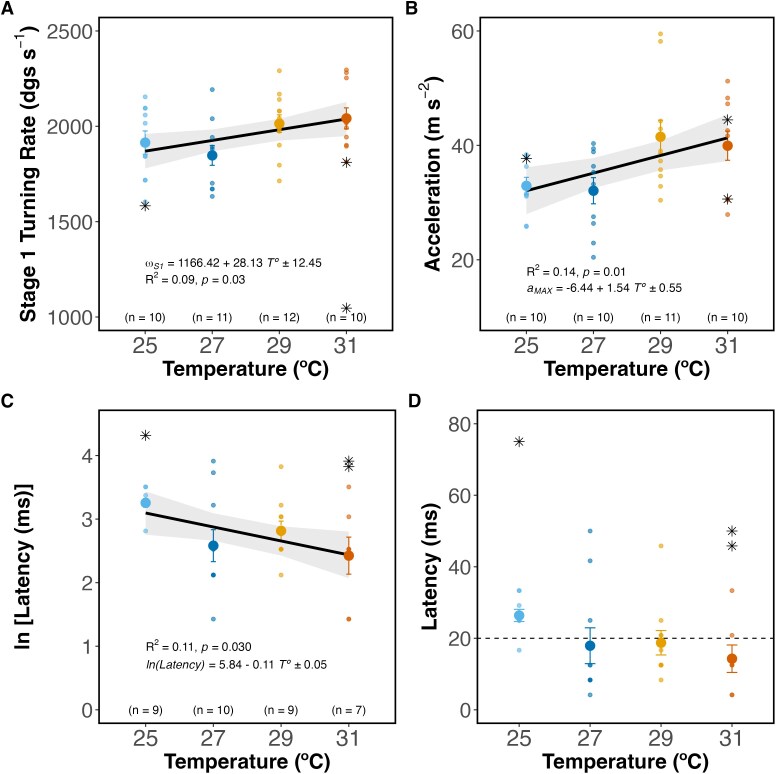
Effect of temperature on escape traits. (**A**) Stage 1 turning rate, (**B**) maximum acceleration and (**C**, **D**) escape latency. Simple linear regressions (black solid line) were fit to double-bend responses only (small dots). Solid circles are means, and bars are standard errors. Bands are 95% confidence intervals. Single-bend responses (*n* = 3) are shown as asterisks but not included in regressions. The dashed line in D marks 20 ms based on the range of latencies observed for mechano-acoustic stimulation (Domenici and Hale, 2019). Sample size is the same for both latency panels.

**Figure 2:**
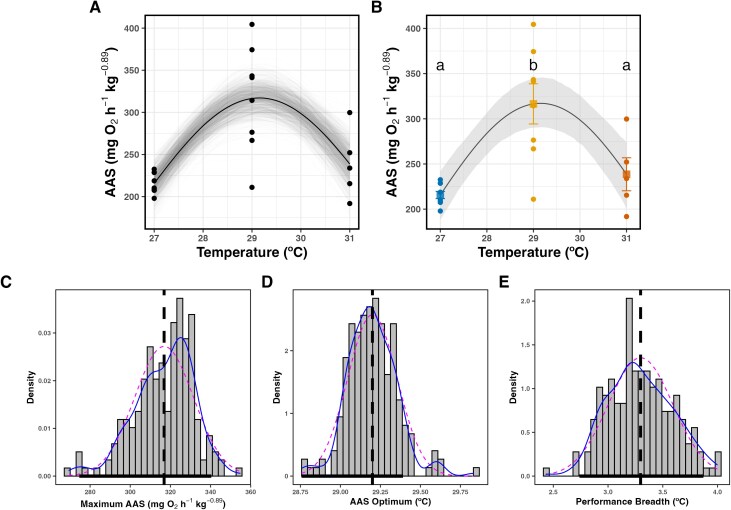
Thermal sensitivity of AAS. (**A**) Residual bootstrapped predictions (grey curves) and best fit (black solid curve). Bootstrapped predictions in (A) were used to calculate model uncertainty in (**B**) as bca 95% CI (grey ribbon in B). Dots are AAS for individual sharks. (B) Best fit (black solid curve) including bca 95% CI (grey ribbon). Different letters indicate statistically significant differences (*P* < 0.05) between means (squares) from pairwise Holm–Sidak *post hoc* tests. Coloured dots are AAS for individual sharks and bars are standard errors around the mean. (**C**–**E**) Bootstrap distributions for derived parameters (vertical dashed lines): maximum AAS, AAS optimum and performance breadth, respectively. Distributions include normal (dashed curve) and kernel (solid curve) density estimates, and bca 95% CI (black horizontal bar).

**Figure 3:**
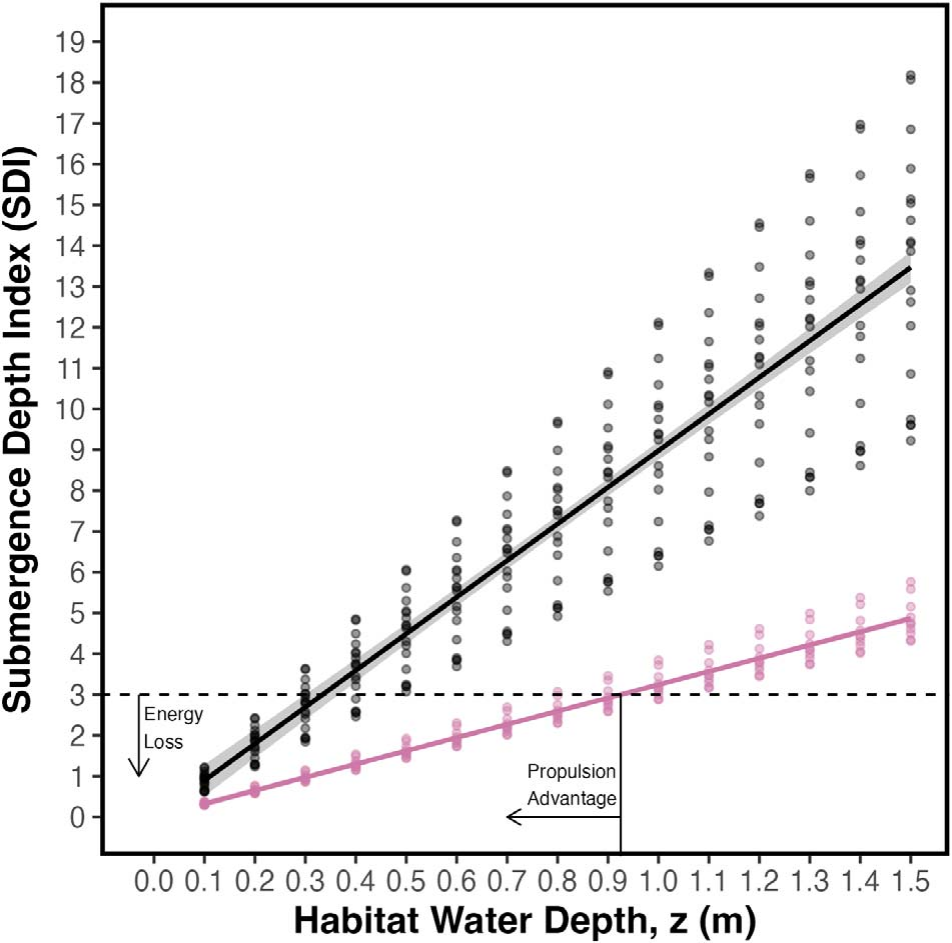
SDI versus habitat water depth. Simple linear regressions (solid lines) were fit to observations (dots) for newborn (black) and adult (pink) blacktip reef sharks. Bands are 95% CIs. Horizontal dashed line marks an SDI = 3, below which drag is significant (thrust loss). The intersection between the regression fits and the SDI limit of 3 shows the habitat water depth at which newborn (0.33 m, left vertical line) and adult (0.92 m, right vertical line) blacktip reef sharks experience significant drag. The area between the vertical lines is considered the depth range where newborns have a propulsion advantage.

**Figure 4:**
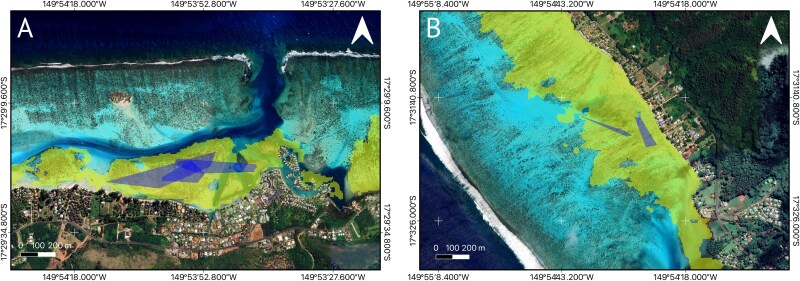
Home range of *C. melanopterus* newborns from Bouyoucos *et al*. (2020). Blue polygons are home ranges for individual sharks obtained from Bouyoucos *et al*. (2020) for (**A**) Tepee and (**B**) Tiki areas. Yellow polygons are extent of the terrestrial reef flat.

**Figure 5:**
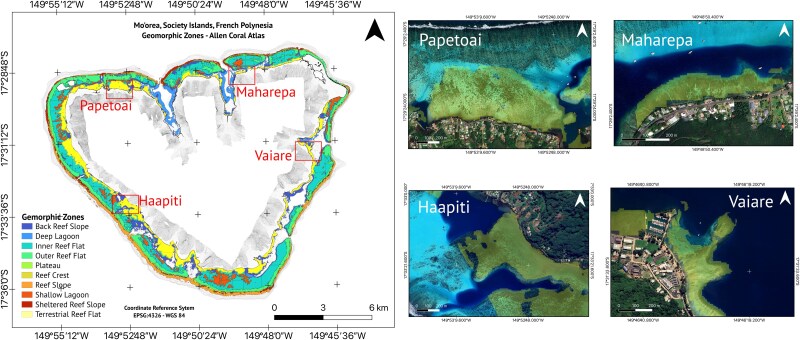
Geomorphic zones for Mo′orea’s reef–lagoon system. Satellite images show extent of the terrestrial reef flats (yellow polygon) in Papetoai, Maharepa, Haapiti and Vaiare. Geomorphic zone polygons are Allen Coral Atlas © 2022 and satellite imagery are Google Satellite ©.

**Figure 6:**
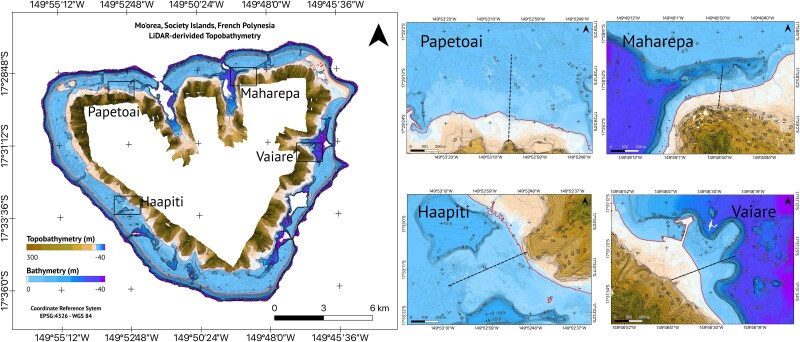
Topobathymetry map for Mo′orea’s reef–lagoon system. Isolines are drawn each 5 m (thicker isolines) and each 0.5 m only <0 m. The coastline is represented by the red isoline at the 0-m elevation level. Black dashed lines represent transects used to build bathymetry profiles (see Fig. 7).

#### Submergence depth index

Submergence depth index (SDI) was calculated following Webb *et al*. (1991) as *z*/*B*, where *z* was the habitat water depth (in metres) and *B* was the shark’s caudal fin height (in metres). We determined *z* as being the habitat water depth instead of the distance from the water surface to the longitudinal dorso-ventral centreline of the sharks because we assume the sharks are swimming close to the bottom in their environment (similar to Webb and colleagues). Additionally, in fish, *B* is the height of their caudal fin as this is the main contributor to thrust (Webb *et al*., 1991). Caudal fin height was measured in a subset of newborn (*n* = 18) and adult (*n* = 10) sharks using caudal fin photographs in ImageJ v2.0.0 (National Institute of Health) as the vertical distance, taken perpendicularly, from the uppermost point of the dorsal lobe to the lowermost point of the ventral lobe of the caudal fin. We calculated SDI at arbitrary habitat water depths ranging from 0.1 to 1.5 m that are expected to be used by newborn *C. melanopterus* (see Bouyoucos *et al*., 2020b). A linear regression was fitted with SDI as the dependent variable, life stage (newborn or adult) as a factor and habitat water depth as the covariate. The difference in slopes was tested via analysis of covariance (ANCThe). The intersection between the fitted lines and an SDI = 3 was used to find the habitat water depth when newborn and adult sharks would experience a reduction in fast-start escape performance. We compared these results with habitat water depth maps and hypothesized that water depth in their nurseries is shallow enough so that drag is significant for predatory *C. melanopterus* adults (i.e. *z*/*B* < 3) but negligible for newborns (i.e. *z*/*B* ≥ 3).

All statistical analyses were performed in the *R* statistical environment (R Core Team, 2019). Visual inspection and Grubbs tests (Grubbs, 1949) were used to check for outliers, and removed when methodological issues were found. Homogeneity of variances was tested with Bartlett’s tests (Bartlett, 1937). An acceptable Type I error rate (α) of 0.05 was used for all regression analyses.

## Results

### Escape performance

All sharks displayed a startle response to all the mechano-acoustic stimulations (i.e. 100% responsiveness) at all the temperatures tested. Startles consisted of a C-shaped, fast-start escape response, always away from the threat. We observed two types of fast-start escape responses: single-bends, where only a first unilateral body bend was observed (Stage 1), and double-bends, where Stage 1 was followed by a contralateral body bend (Stage 2). When all responses were considered for each individual (three startles each), we observed that 3.7 and 7.4% of all the responses at the extreme cold (25°C) and warm (31°C) temperatures, respectively, were single-bends. No single-bends were reported at the intermediate temperatures of 27 and 29°C. We used only double-bends to examine escape performance to avoid differences due to response type.

At higher body temperatures, sharks had higher maximum turning rates (Fig. 1A; LM, effect of temperature, *F*_(1, 41)_ = 5.12, *P* = 0.03) and maximum accelerations (Fig. 1B; LM, effect of temperature, *F*_(1, 39)_ = 7.73, *P* = 0.01) but similar maximum speeds (LM, effect of temperature, *F*_(1, 42)_ = 2.15, *P* = 0.15). Sharks also reacted faster to a stimulus at higher temperatures (Fig. 1C; LM, effect of temperature, *F*_(1, 34)_ = 5.14, *P* = 0.03, for log transformed latency). Sharks reacted 12.1 ms faster at 31°C compared to 25°C (Fig. 1D). At 25°C, response times ranged from 16.70 to 33.30 ms (26.4 ± 1.7 ms; mean ± SE hereafter). At 27°C, the fastest response was 4.16 ms, and the slowest was 50.00 ms (17.9 ± 5.0 ms). At 29°C, responses ranged from 8.33 to 45.80 ms (18.75 ± 3.4 ms), while at 31°C, they ranged from 4.16 to 33.30 ms (14.3 ± 3.8 ms).

### Aerobic scope performance

Three sharks from the 27°C group, four from the 29°C group and four from the 31°C group were not included in the metabolic rate analyses due to the three main reasons explained below. Firstly, one individual from the 27°C group, three from the 29°C group, and three from the 31°C group were removed from analysis because these sharks remained in a bent position inside the chambers throughout the trial. Secondly, one more individual from the 29°C group showed loss of equilibrium during respirometry and was removed from the chamber, returned to the holding tank, and allowed to recover before being released. Thirdly, another two individuals died during respirometry, one from the 29°C group, and one from the 31°C group. Therefore, the final sample sizes for each temperature were as follows: 27°C (*n* = 9), 29°C (*n* = 8) and 31°C (*n* = 8).

Maximum oxygen uptake rates (*Ṁ*_O2Max_) increased from 27 to 29°C, but decreased from 29 to 31°C, and minimum oxygen uptake rates (*Ṁ*_O2Min_) increased from 27 to 31°C (see Fig. S3). A Gaussian function provided the best fit to the AAS, outperforming the quadratic model by 9.45 AICc points and showing relatively good certainty (Fig. 2A and B). Fitted curves showed AAS peaked at 317.10 mg O_2_ h^−1^ kg^-0.89^ (bca 95% CI [274.10–341.40]) at 29.15°C (bca 95% CI [28.80–29.40]; Fig. 2C). Modelled AAS dropped <80% at temperatures <27.5°C and >30.8°C. Therefore, based on the modelled trend, the breadth to maintain high aerobic scope performance is 3.30°C (bca 95% CI [2.80–3.90]; Fig. 2C). This trend in AAS should be interpreted with caution because modelled outputs were informed by only three measured temperatures. Notably, pairwise Holm–Sidak *post hoc* tests confirmed statistically significant differences in mean AAS between 27 and 29°C, and between 29 and 31°C, but not between 27 and 31°C (Fig. 2B).

### Submergence depth index

Caudal fin heights spanned 31.11 ± 0.95 cm in adult and 11.66 ± 0.62 cm in newborn blacktip reef sharks. Newborn sharks had a higher overall SDI than adult sharks (LM, effect of life stage, *F*_(1,416)_ = 1079.1, *P* < 0.05; Fig. 3). Drag was predicted to be significant (i.e. SDI ≥ 3) at water depths ≤0.92 m in adult blacktip reef sharks, but at water depths ≤0.33 m in newborns. That is, newborn blacktip reef sharks are predicted to have a fast-start propulsive advantage within a range of depths of 0.33–0.92 m.

### Habitat features

Mo′orea’s reef–lagoon system encompassed ~50.63 km^2^ and presented 10 out of 12 geomorphic classes (Figs. S1 and Table S1). The largest geomorphic class was the inner reef flat, with 15.05 km^2^, ~29.72% of the entire reef–lagoon area (Table S1). The areas where *C. melanopterus* newborns have been observed, as reported in previous studies using mark-recapture and active acoustic tracking data, correspond to a single-habitat morphotype: the terrestrial reef flat (Figs 4 and 5). Terrestrial reef flats around Mo′orea extended 8.02 km^2^, along the coast, and covered 15.85% of the reef–lagoon system.

**Figure 7:**
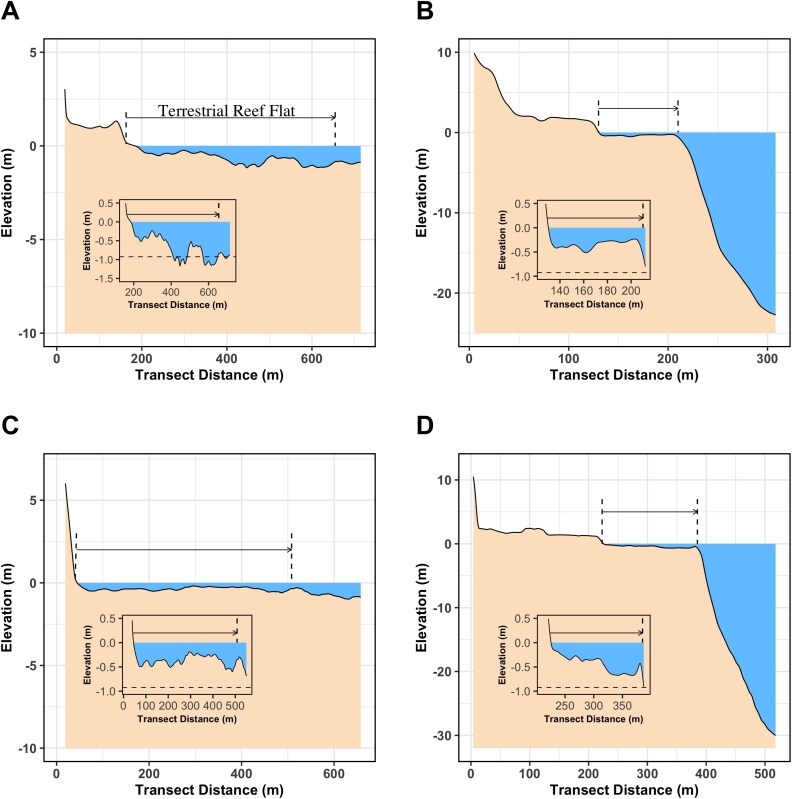
Bathymetry profiles. Profiles for (**A**) Papetoai, (**B**) Maharepa, (**C**) Haapiti and (**D**) Vaiare. The horizontal arrows show the terrestrial reef flat extent measured from the coastline (0 m). (Inserts) Zoomed profiles showing the limit depth for propulsive advantage (horizontal dashed lines). Waters shallower than this line provide a propulsive advantage to newborn blacktip reef sharks over adult conspecifics.

**Figure 8:**
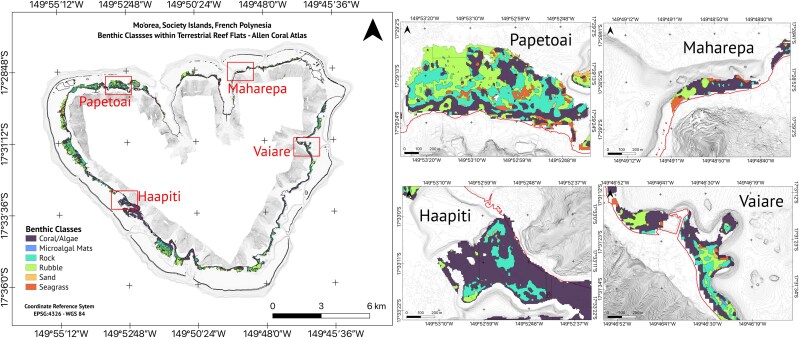
Benthic classes within the terrestrial reef flat around Mo′orea. Benthic class polygons are Allen Coral Atlas © 2022. The coastline is represented by the red isoline at the 0-m elevation level.

The terrestrial reef flat had a median water depth of 0.74 m (Fig. 6 and Table 1). This water depth was shallower than the limit for propulsive advantage (0.92 m) in newborn blacktip reef sharks (Figs 3 and 7). The benthic habitat within the entire terrestrial reef flat around Mo′orea was primarily composed of coral/algae (43.3%) and rubble (28.1%) and rock (21.5%), followed by seagrass (2.98%), sand (2.80%) and microalgal mats (0.08%) (Fig. 8). Our *in situ* observations validated that the coral/algae class consisted mainly of coral (Fig. S2). However, many coral colonies were found to be dead and covered with macroalgae (Fig. S2B).

**Figure 9:**
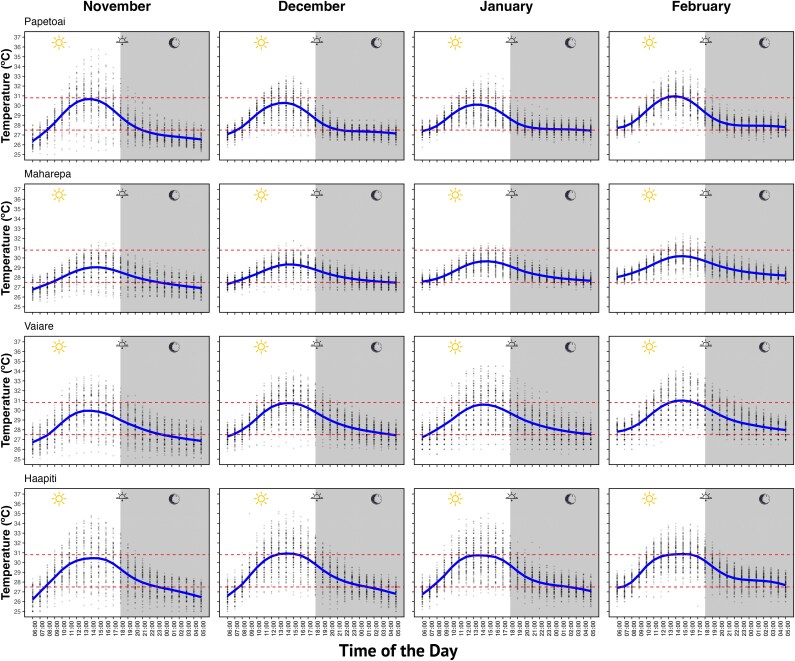
Historical 24-h thermal regimes. Continuous blue lines are LOESS smooths of the temperature recorded at each hour of the day (grey dots) across four consecutive seasons: 2019–2020, 2020–21, 2021–22 and 2022–23. The estimated range for maintaining ≥80% of peak aerobic scope in *C. melanopterus* is indicated between 30.8 and 27.5°C (red dashed lines).

Temperature LOESS trends estimated a clear diurnal temperature pattern across all sites and months, with temperatures increasing during the daytime, peaking around midday to early afternoon and decreasing during the evening and night-time (Fig. 9). The mean habitat temperature was 28.61 ± 0.09°C, with a daily thermal range of 3.20 ± 0.21°C, and minimum and maximum temperatures of 2.22 and 30.34 ± 0.14°C, respectively (when all sites were averaged, Table 2). Maximal habitat temperatures occurred between 13 h00 and 15 h00 but decreased afterwards, reaching minimum temperatures at 06 h00 (Fig. 9 and Table 2). Temperatures largely aligned within the range identified in laboratory tests for sustaining ≥80% of peak aerobic scope performance in *C. melanopterus* (dashed lines in Fig. 9). However, maximal temperatures frequently reached or exceeded the upper limit between 13 h00 and 15 h00 (red).

All habitats were thermally volatile with a tendency to be warmer than the estimated trend, particularly between 12 h00 and 17 h00, but became more thermally stable after sunset (18 h00–18 h30) until early morning hours (06 h00–08 h00; Fig. S4). November was the most thermally volatile month, and February the most thermally stable month. Maharepa was the most thermally stable habitat across all 4 months. The period of higher volatility coincided with the time at which we recorded the maximum habitat temperatures. This explains why we recorded temperatures of up to 36°C (grey dots in Fig. 9) with daily ranges as large as 10°C in some cases (e.g. in Papetoai and Haapiti). That is, we recorded events when the temperature within the terrestrial reef flats exceeds by ~6°C the estimated maximum habitat temperature.

## Discussion

Our findings support the hypothesis that shark nurseries can reduce post-encounter predation risk by providing environmental conditions that maximize escape performance. Consistent with this hypothesis, we demonstrated that the thermal conditions within Mo′orea’s nurseries optimize reaction times, fast-start acceleration and turning rates. Additionally, the shallow water depth in *C. melanopterus* nurseries (median depth: 0.74 m) may impair the propulsive abilities of adult conspecifics (i.e. potential predators), giving newborns a hydrodynamic advantage during predator encounters. The nurseries also feature predominantly coral substrates, a structurally complex habitat that enhances escape potential by leveraging the sharks’ exceptional turning performance (Webb, 1976; Trujillo *et al*., 2022). Finally, we showed that the thermal conditions in these nurseries support aerobic scope performance at ≥80%, likely facilitating post-exercise recovery and further improving survival outcomes for *C. melanopterus* newborns.

### Fast-start thermal response

The observed positive correlations between fast-start acceleration, turning rate and reaction time with temperature align with previous findings on the thermal effects of escape responses in teleost fishes (Johnson and Bennett, 1995; Domenici *et al.*, 2019), particularly in relation to acute temperature changes. Muscle performance and power output tend to increase with acute warming, as seen in other species (Wakeling, 2005; James, 2013), which explains the increase in fast-start acceleration and turning rate at warmer temperatures in *C. melanopterus* newborns. This increase in acceleration and turning rate, but not in speed, is likely attributable to the acute response of myofibrillar ATPase activity and twitch contraction kinetics to acute temperature changes, which increase with acute warming (Johnson and Bennett, 1995). This fast muscular response provides them with the ability to generate rapid tangential acceleration, increasing the chances of newborn sharks to evade predator attacks (Walker *et al.*, 2005). Moreover, the fast reaction times evidence the effect of acute thermal exposure on neural conduction speeds, which usually yield shorter latencies at warmer temperatures (Szabo *et al.*, 2008; Penghan *et al.*, 2016). Sharks reacted 1.8 times faster at warmer temperatures (i.e. 12 ms faster). This represents a significant improvement in reaction time, considering that survival during a predator strike can often depend on just a few milliseconds (McCormick *et al.*, 2018).

**Table 1:** Habitat water depths for each of the geomorphic zones found in Mo′orea

		Depth (m)	
Geomorphic zone	Extent (km^2^)	Median	Variance
Back reef slope	4.67	8.83	6.44
Deep lagoon	3.13	13.28	11.54
Inner reef flat	15.05	1.42	0.09
Outer reef flat	9.61	1.31	0.85
Plateau	0.03	7.55	3.41
Reef crest	1.35	0.67	0.55
Reef slope	3.89	6.13	6.10
Shallow lagoon	3.34	1.83	0.16
Sheltered reef slope	1.53	4.07	1.83
Terrestrial reef flat	8.02	0.74	1.51

Water depths were calculated as the median water depth within the corresponding polygon (see Fig. 5) obtained from the bathymetric maps (see Fig. 6). Notice that Reef Crest is a boundary zone between the Reef Flat and the Reef Slope unlikely to be available for newborn blacktip reef sharks.

Although we did not observe a significant decline in fast-start escape performance at the upper end of our temperature range (31°C), we believe that the optimal temperature for fast-start escape responses lies within the range of temperatures used in our study, and not beyond 31°C. For instance, the predicted turning rates were faster than expected for their body size (Domenici, 2001) at all temperatures. According to Trujillo *et al*. (2022), a newborn blacktip reef shark should attain a 783-degree.s^−1^ turning rate. We predicted much faster turning rates, of 2038 degree.s^−1^ (95% CI [1948–2128]) at 31°C, and >1700 degree.s^−1^ for the lower temperatures. Similar to turning rate, average escape latencies were not only within the most commonly observed range of latencies in response to a mechanical stimulus (i.e. 5–20 ms, see Domenici and Hale, 2019), but we also recorded reaction times as fast as 4.16 ms between 27 and 31°C. These reaction times likely approximate the fastest escape latencies that can possibly be evoked mechanically (see Tabor *et al*., 2014). Although maximal fast-start escape responses likely lie within the range of temperatures we tested, we also observed a behavioural response to changes in temperature: we noticed a higher occurrence of single-bends at 25 and 31°C, with no instances of single-bends recorded at 27 and 29°C. The temperature at which single-bends occur coincides with the temperature at which AS declines. Single-bends are considered less energetically costly than double-bend responses (Domenici and Hale, 2019). As such, previous studies have found single-bends to be more frequent during physiological challenges (Lefrançois *et al*., 2005) like starvation (Yan *et al*., 2015) and hypoxia (Domenici *et al*., 2007). Although 25 and 31°C may not pose a physiological challenge to *C. melanopterus* newborns (but see Bouyoucos *et al*., 2020a), their aerobic scope drops <80% at these temperatures, suggesting that the occurrence of single-bends at these temperatures reflect a compromise between conserving energy and maintaining performance under these conditions. While subtle, these results suggest that optimal fast-start escape responses in blacktip reef sharks are achieved between 27 and 29°C, with behavioural adjustments evident at 25 and 31°C.

While long-term acclimation may compensate the effects of temperature on fish escape performance, acute temperature changes are likely more critical for this shark population due to the significant short-term temperature variability they experience (Domenici *et al*., 2019). In our experiments, sharks were initially exposed to a natural daily temperature regime ranging from 25 to 32°C (a 7°C range). We then cooled or heated the water at a rate of 0.5°C day^−1^ for 3 days before reaching the experimental test temperatures. In the wild, newborn sharks may face much more abrupt temperature fluctuations, with daily maximum heating rates ~1.4°C h^−1^ (Bouyoucos *et al*., 2021) and daily temperature changes of up to 10°C (see Fig. 9). Moreover, we observed that the range of temperatures to achieve high aerobic scope (i.e. ≥80%) is much narrower than the temperatures in which they operate (T_b_ = 26.1–34.1°C, Bouyoucos *et al*., 2020a). This often results in exposure beyond their upper limit for optimal aerobic scope, rendering them susceptible to extreme temperature events. For instance, temperatures exceeding 31°C may impair their reaction to predators due to alterations to their neurosensory system (Webb and Zhang, 1994). Additionally, sharks may struggle to perform double-bend responses, instead relying on the less powerful single-bend fast-start escape responses. Alternatively, to avoid extreme heat, sharks may retreat to deeper waters, inadvertently increasing their exposure to predators. However, previous tracking studies suggest these sharks do not leave the terrestrial reef flats. This behaviour may be attributed to their thermal inertia (Papastamatiou *et al*., 2015), which enables them to maintain lower body temperatures for a few hours after heat exposure. Whether they stay in or leave the flats, temperature plays a critical role modulating their predation risk, far more so than tides, as observed in other nursery systems (Guttridge *et al*., 2012). Consequently, future research should investigate the spatial behaviour and mortality rates of newborn sharks in relation to temperature, particularly during prolonged warming events.

We found no evidence of neural impairment in response to acute thermal exposure during mechano-acoustic stimulation. Responsiveness remained at 100%, with all turns away from the threat, contrary to findings from acclimation studies where temperatures approached the fish’s thermal thresholds (Szabo *et al*., 2008). Nonetheless, temperature-induced effects on vision could still impair a fish’s ability to detect and anticipate predator attacks, potentially reducing survival even before neural impairment occurs. For instance, fish escape responses can also be triggered visually by the magnifying retinal image of an approaching object (i.e. the looming effect, Domenici, 2002). Apparent looming threshold (ALT), the reaction distance of a startled fish proportional to the predator’s speed and frontal profile (Dill, 1974), may increase with acute warming. Increased ALT due to high-temperature exposure means reduced reaction distances despite high predation threat (Allan *et al*., 2015) and have been shown to increase prey vulnerability to predators (Webb and Zhang, 1994). Because we used a sudden mechano-acoustic stimuli, we are unable to determine whether vision was impaired at the temperatures to which the sharks were exposed. Therefore, further investigations using looming visual stimuli could shed light on the effects of acute warming on the sensory performance of newborn blacktip reef sharks.

**Table 2:** Twenty-four-hour thermal regimes for each site per month

		24-h Thermal regime parameters					
Site	Month	Mean T°C	Minimum T°C	Time of Minimum	Maximum T°C	Time of Maximum	Range °C
Papetoai	November	28.21	26.41	06 h00	30.62	14 h00	4.21
	December	28.33	27.01	06 h00	30.28	14 h00	3.27
	January	28.41	27.33	06 h00	30.10	13 h00	2.77
	February	28.91	27.71	06 h00	30.96	14 h00	3.25
Maharepa	November	27.91	26.85	06 h00	29.12	15 h00	2.27
	December	28.25	27.40	06 h00	29.34	15 h00	1.94
	January	28.49	27.57	06 h00	29.65	15 h00	2.08
	February	29.02	28.04	06 h00	30.18	15 h00	2.14
Vaiare	November	28.27	26.69	06 h00	29.99	14 h00	3.30
	December	28.88	27.31	06 h00	30.73	14 h00	3.43
	January	28.83	27.34	06 h00	30.58	14 h00	3.24
	February	29.25	27.85	06 h00	31.00	15 h00	3.15
Haapiti	November	28.39	26.22	06 h00	30.44	14 h00	4.23
	December	28.74	26.59	06 h00	30.92	14 h00	4.33
	January	28.72	26.80	06 h00	30.69	14 h00	3.89
	February	29.10	27.41	06 h00	30.86	15 h00	3.45

Values are obtained from the LOESS fits for each month and site.

### Habitat features

With a median depth of 0.74 m, *C. melanopterus* nurseries are shallow enough to hinder the propulsive capacity of adult blacktip reef sharks. Shallow water has been shown to limit the swimming capacity of large fish (Bourne *et al*., 2011; Shiau *et al*., 2020). Since predators rely on fast-starts to strike prey (Harper and Blake, 1991; Domenici and Blake, 1997), newborn blacktip reef sharks may gain an advantage when escaping predator attacks within Mo′orea’s terrestrial reef flats. Furthermore, because shallow water disproportionately impacts larger fish (Webb *et al*., 1991; also see Domenici, 2001), adult blacktip reef sharks are likely to experience greater thrust reduction during strikes compared to their smaller conspecific targets performing fast-start escape responses in these shallow habitats. During the propulsive stage of a predator’s strike (Schriefer and Hale, 2004), much of the energy is transferred into surface waves (Hertel, 1966) rather than forward thrust when striking in shallow water (e.g. see supplementary video 1). Ground effects are unlikely to offset the drag costs experienced by predators striking prey in shallow waters. This is because such effects are most relevant for dorsoventrally compressed fish (Blevins and Lauder, 2013; Su *et al*., 2022) and are typically observed during low-acceleration, high-velocity manoeuvres (Quinn *et al*., 2014). Consequently, ground effects are not significant during a forward fast-start strike, which involves high accelerative manoeuvres (Schriefer and Hale, 2004). Finally, the rapid turning rates we observed in newborn sharks, coupled with their ability to execute sharp turns with small turning radii (as recorded by Trujillo *et al*., 2022), highlight their remarkable manoeuvrability (Webb, 2004). This turning performance likely provides a key advantage in outmanoeuvring larger predators, as turning performance declines with increasing body size (Domenici, 2001). Moreover, this trait aids navigation through the complex, coral-dominated flats that are characteristic of Mo′orea’s terrestrial reef flats (Domenici and Blake, 1997; Blake, 2004).

### Aerobic scope thermal performance

We established a correlation between aerobic scope and temperature in *C. melanopterus* newborns, providing insights for models that link organismal physiology to habitat suitability (e.g. see Del Raye and Weng, 2015; Teal *et al*., 2018). The temperature for peak aerobic scope (29.1°C) closely aligns with the average body temperatures (29.6 ± 1.2°C) at which these newborns operate around Mo′orea (Bouyoucos *et al*., 2020a), suggesting that the optimum temperature for aerobic scope (T_opt_) is near 29°C. While our modelled estimates are consistent with observed body temperatures in the wild and with the hypothesized T_opt_ for the species (as suggested by Bouyoucos *et al*., 2020a), uncertainty remains about how AAS responds outside the limited range of three tested temperatures. If our estimates are accurate, the high AAS at ~29°C likely supports the recovery capacity from exercise such as that due to escaping from predator attacks in newborn *C. melanopterus* sharks (Marras *et al*., 2010). While a thermal performance curve incorporating additional temperatures would provide greater resolution, our findings highlight the critical role of aerobic scope in shaping thermal preferences, particularly under daily thermal fluctuations.

Daily thermal fluctuations, driven by local meteorological changes such as solar radiation (Leichter *et al*., 2006) and cloud cover (Leahy *et al*., 2013), can increase Mo′orea’s reef flats water temperatures by ~6°C above the average maximum, occasionally reaching up to 36°C. Such extremes surpass the upper thermal limit (CT_Max_ = 35.9 ± 0.4°C; Bouyoucos *et al*., 2021) and safety margin (TSM ~6°C; Bouyoucos *et al*., 2021) of newborns, leaving them reliant on behavioural strategies to avoid overheating (Sunday *et al*., 2014). Indeed, it is possible that these sharks lack a physiological thermal safety margin (Parmesan *et al*., 2000; Parmesan, 2006). For example, newborns actively avoid temperatures ≥31°C (Bouyoucos *et al*., 2020a), likely because aerobic scope drops <80% at these temperatures. This avoidance behaviour underscores the thermal dependence of aerobic scope and suggests that these sharks employ behavioural strategies to mitigate exposure to potentially harmful thermal conditions.

Although the *C. melanopterus* population around Mo′orea appears relatively robust to ocean warming (Bouyoucos *et al*., 2020a), extreme temperature events such as marine heatwaves (e.g. marine heatwaves; Holbrook *et al*., 2019; Smale *et al.*, 2019) could intensify daily thermal extremes and compromise their ability to exploit critical habitats that help reduce predation risk. Sustained heating exposure can also prolong post-exercise metabolic recovery times (Zeng *et al*., 2010), with Bouyoucos *et al*. (2018) extrapolating recovery durations of 3.1–19.8 h following gill-net capture and air exposure. Accurate estimates of metabolic recovery costs, particularly the duration of excess post-exercise oxygen consumption (EPOC), are essential to understanding the energy demands of repeated anti-predator responses in this population. Marine heatwaves, already documented around Mo′orea, intensify heating effects in local shallow ecosystems (Wyatt *et al*., 2023). Newborn *C. melanopterus* already operate within body temperatures (26.1–34.1°C, Bouyoucos *et al*., 2020a) that approach or exceed the upper threshold our model estimates is required to maintain aerobic scope >80% (i.e. 30.8°C). However, this estimate should be interpreted with caution given it is derived from a model fit to only three tested temperatures. If the estimated trend holds, which is consistent with patterns reported in other nursery bound elasmobranchs (Lear *et al*., 2019), it could explain their strong behavioural tendency to avoid temperatures exceeding 31°C (Bouyoucos *et al*., 2020a). Avoidance of these habitats may expose them to predators (Guttridge *et al*., 2012,), and limit opportunities for acclimation responses (Johnson and Bennett, 1995; Da Silva *et al*., 2019), particularly if mortality rates increase significantly. These findings underscore the importance of understanding both physiological and behavioural responses to warming events to predict the resilience of shark population in a rapidly changing climate.

#### Mortalities during respirometry

The two mortalities observed at 29 and 31°C may suggest reduced ATP synthesis efficiency at supraoptimal temperatures (i.e. temperatures exceeding the Arrhenius break temperature, ABT; Sokolova, 2023), leading to an expanded aerobic scope and an unreliable estimation of the temperature for peak AAS. However, heat-induced mitochondrial damage is unlikely to be a proximal cause of mortality in ectotherms (Chung and Schulte, 2020; Sokolova, 2023). Instead, mitochondrial efficiency—specifically, that balance between ATP production and energy expenditure—was expected to remain optimal within the tested temperature range (i.e. below ABT), supporting the validity of our estimates. Mortality is more likely attributed to post-exercise intracellular acidosis (Wood *et al*., 1983), potentially exacerbated by stress responses to respirometry chambers at elevated temperatures (Bouyoucos *et al*., 2018; Bouyoucos *et al*., 2020a).

### Habitat-specific escape advantage in shark nurseries

Our study shows that Mo′orea’s terrestrial reef flats provide environmental conditions that may reduce post-encounter predation risk. Normally, shark nurseries are believed to be separated from adult habitats (Beck *et al*., 2001) to reduce pre-encounter predation risk (Clarke, 1971; Branstetter, 1990; Guttridge *et al*., 2012). However, spatial overlap between predators and prey does not necessarily indicate the level of predation risk (Suraci *et al*., 2022). We established that *C. melanopterus* newborns likely have a habitat-specific escape advantage within their nurseries, the terrestrial reef flats, where they are able to achieve maximal escape performance. Maximal fast-start escape responses are vital to maximize the chances of survival following encounters with predators (e.g. McCormick *et al*., 2018; Domenici and Hale, 2019), particularly for small juveniles (see introduction). Therefore, the anti-predator strategy used by *C. melanopterus* newborns appears to be focused on facilitating successful escapes from predator encounters instead of solely minimizing the likelihood of encountering predators (e.g. Camacho, 2014). This habitat-specific escape advantage complements established attributes that make nearshore coastal habitats important as shark nursery areas, such as ample food supplies, predator exclusion and conditions that facilitate growth.

## Supplementary Material

Web_Material_coaf045

## Data Availability

The data underlying this article will be shared on reasonable request to the corresponding author.
