# Supplementary material for: Safety in the shallows: nearshore coastal habitats can provide physical and thermal features that optimize escape performance in newborn blacktip reef sharks (Carcharhinus melanopterus)
Source: Conserv Physiol. 2025 Aug 4;13(1):coaf045. doi: 10.1093/conphys/coaf045 (PMC12320776; doi:10.1093/conphys/coaf045)
Supplement: Web_Material_coaf045 [file web_material_coaf045.zip › Supplementary_material_safety_in_the_shallows_Conservation_Physiology_Trujillo_et_al_FINAL.pdf]

Supplementary material accompanying the research article entitled: **Safety in the shallows: nearshore coastal habitats can provide physical and thermal features that optimize escape performance in newborn tropical reef sharks**

José E. Trujillo[1,*], Ian A. Bouyoucos[2,3], Ornella C.Weideli[3,4,5], Elena M.C. Milanesi[6], Shamil F. Debaere[7,8], William J. Rayment[1], Serge Planes[3,8], Paolo Domenici[9,10], Jodie L. Rummer[2,8], & Bridie J.M. Allan[1]

[1]Department of Marine Science, University of Otago, New Zealand

[2]Australian Research Council Centre of Excellence for Coral Reef Studies, James Cook University, Australia

[3]PSL Research University, EPHE-UPVD-CNRS, USR 3278 CRIOBE, Université de Perpignan, France

[4]Private University in the Principality of Liechtenstein (UFL), Triesen, Principality of Liechtenstein

[5]Dr Risch Medical Laboratory, Wuhrstrasse 14, 9490 Vaduz, Liechtenstein

[6]Department of Earth and Environmental Sciences (DISAT), University of Milano - Bicocca, Piazza della Scienza, 1, Milano 20126, Italy

[7]ECOSPHERE, Department of Biology, University of Antwerp, Groenenborgerlaan 171, 2020 Antwerp, Belgium

[8]Marine Biology, College of Science and Engineering, James Cook University, Australia

[8]Laboratoire d'Excellence "CORAIL", EPHE, PSL Research University, UPVD, CNRS, USR 3278 CRIOBE, French Polynesia

[9]CNR-IAS, Località Sa Mardini, 09170, Torregrande, Oristano, Italy

[10]CNR-IBF, Area di Ricerca San Cataldo, Via G. Moruzzi N°1, 56124, Pisa, Italy

*Corresponding author: José E. Trujillo (josemiliotrujillo@gmail.com)

Methods

Escape trial

Individual sharks were randomly sampled from their holding tanks, adjacent to the test arena, with hand nets and transferred to the test arena, which was completed in less than 30 s. To restrict movement in the vertical plane, water was maintained at a depth of 16-19 cm, which just allowed the whole body of the shark to be submerged. The mechano-acoustic stimulus consisted of a tapered steel weight (560 g) that was released by an electromagnet from 1.58 m above the test arena onto the surface of the water through a PVC pipe held until 3 cm before to avoid a premature response (Allan et al., 2014; Dadda et al., 2010). A mirror positioned close to the water surface at 45º to the plane of the camera allowed us to record the onset of the stimulation (Marras et al., 2011). Each response was filmed at high speed (240 frames s^{-1}) using a GoPro Hero7 Black camera fixed above the test arena and facing vertically down. Three sharks were tested per day for any given temperature using natural lighting. Tests always took place after 07h00 and were complete before 18h00 to control for diel changes in behaviour. We used three startles given that individuals are likely to vary in their performance across multiple stimulations, the strongest not necessarily being the first response (Marras et al., 2011). Trujillo et al., (2022) detected no evidence of fatigue after three similar startles. Sharks were only startled when they were within the target zone.

Respirometry

We used purpose-build respirometry chambers (~32 L) to measure oxygen uptake rates ($\dot{M}_{O2}$). Three chambers were placed in a water bath at the corresponding treatment temperature such that the three sharks coming from the escape trial were tested in the same group (one per chamber). A flush pump was controlled by a digital relay timer set to cycles of 10 minutes on (flush period) and 5 minutes off (O_2 measuring period). Individual recirculating pumps connected in a loop to the chambers allowed water mixing within each chamber. We ensured O_2 did not drop below 80% saturation during the measuring period.

Dissolved oxygen concentration (DO, in mg l^{-1}) was measured with fibre optic probes inserted into each chamber through the overflow outlets and connected to a FireSting Optical Oxygen Meter (PyroScience GmbH, Aachen, Germany). We used a single temperature probe placed in the water bath to allow for temperature-corrected DO readings in addition to barometric and salinity corrections. Oxygen probes were calibrated to 100% air saturation before each trial and to 0% with a sodium sulphite solution as needed (Rummer et al., 2016).

Background respiration was estimated using the same, empty chambers for 1 h before and after testing sharks (Rummer et al., 2016).

The DO measurements were used to calculate $\dot{M}_{O2}$ (in mg O_2 h^{-1} kg^{-1}) using the formula:

$$\dot{M}_{O2} = SV_{resp} M^{-1},$$

where S is the slope of the linear decline in DO (in mg O_2 l^{-1} s^{-1}) with a coefficient of determination > 0.95, V_{resp} is the volume of the water in the respirometry chamber (32 L, not including the shark), and M is the mass of the shark (in kg). Measurement cycles yielded 96 $\dot{M}_{O2}$ determinations in 24 hours. Values of S were calculated using the custom R code *RespiroRS* (A. Mercière and T. Norin unpublished data, https://github.com/Alexmerciere/RespiroRS.git).

Figures

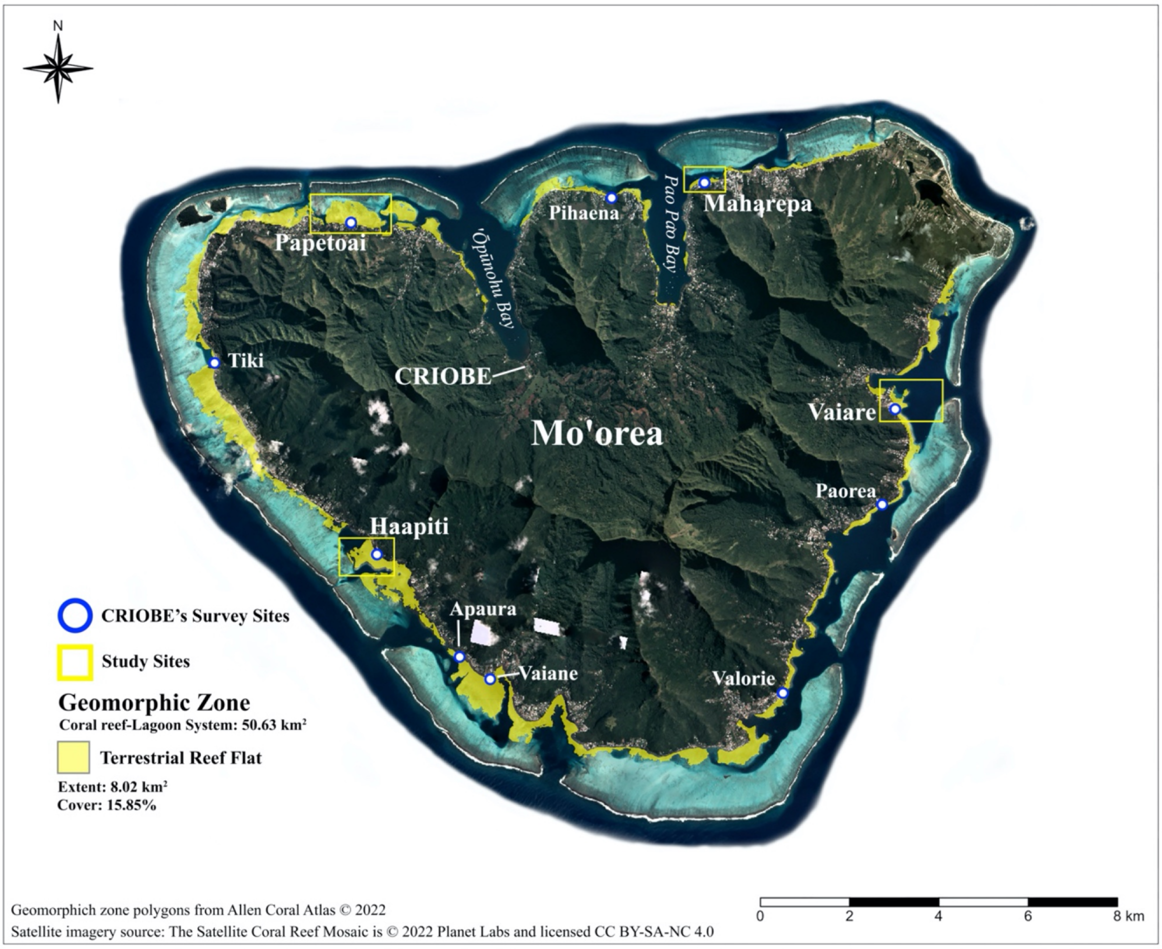

Figure S1. Study areas and terrestrial reef flats around the island of Mo'orea. Satellite view of Mo'orea showing the study sites (yellow rectangles) as it relates to CRIOBE's 10 survey sites (blue-white circles) and the extent of the terrestrial reef flats along the coast (yellow area). Sharks used for experiments were collected from Papetoai (n = 16), Maharepa (n = 8), Vaiare (n = 10), Valorie (n = 1), Haapiti (n = 2) and Tiki (n = 11). Geomorphic zone polygons and satellite imagery from the Allen Coral Atlas © 2022 (see map for complete credit) were processed and plotted with EPSG:4326 geodetic coordinate refence system using the *sf*, *raster* and *tmap* R packages. (B) Aerial view of the study sites: Papetoai, Maharepa, Vaiare and Haapiti.

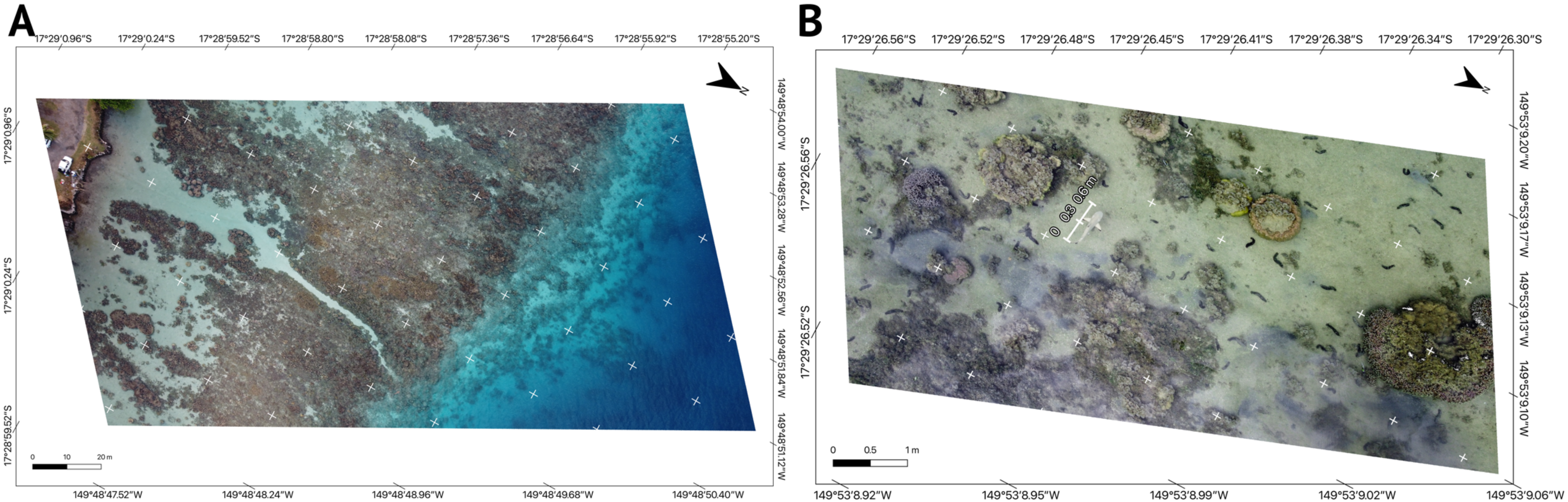

Figure S2. Georeferenced nadir photographs from an unmanned aerial vehicle (UAV). (A) Coral cover in Maharepa. (B) A newborn blacktip reef shark swimming between coral colonies in Papetoai. Notice some coral colonies are dead and covered with macroalgae in B.

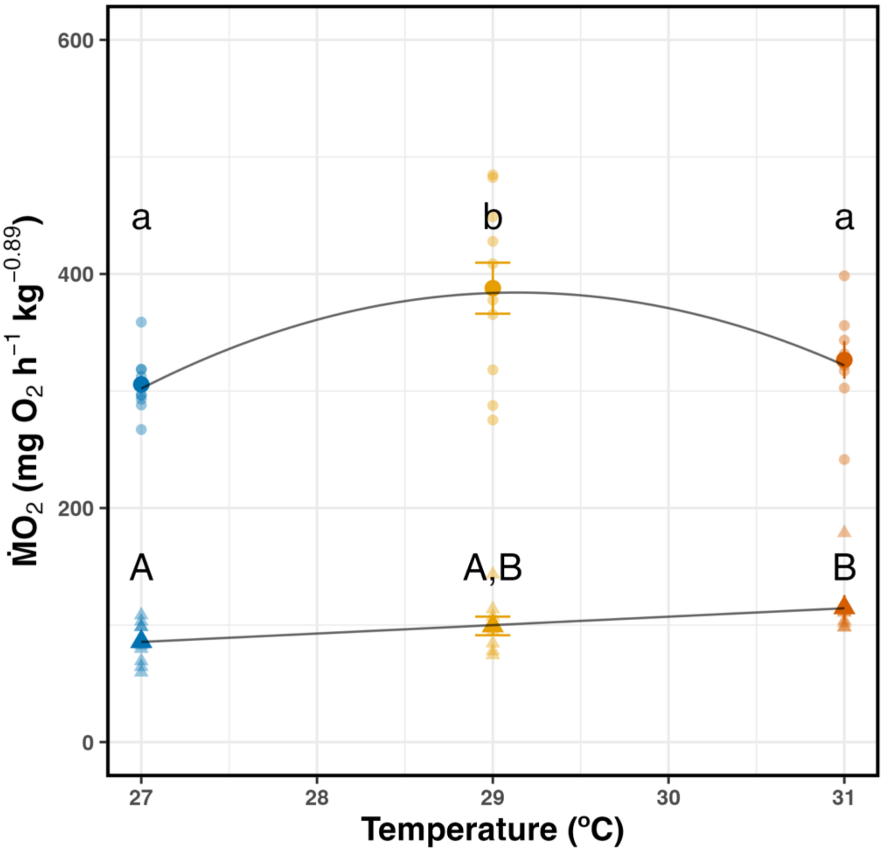

Figure S3. Effect of temperature on maximum ($\dot{M}_{O2Max}$) and minimum ($\dot{M}_{O2Min}$) oxygen uptake rates. Different letters indicate statistically significant differences ($p < 0.05$) between means (solid circles and triangles) from pair-wise Holm-Sidak *post hoc* tests. Small dots and triangles are observations and bars are standard errors. Fitted lines are second degree and first-degree polynomials for $\dot{M}_{O2Max}$ ($R^2 = 0.30$) and $\dot{M}_{O2Min}$ ($R^2 = 0.22$), respectively.

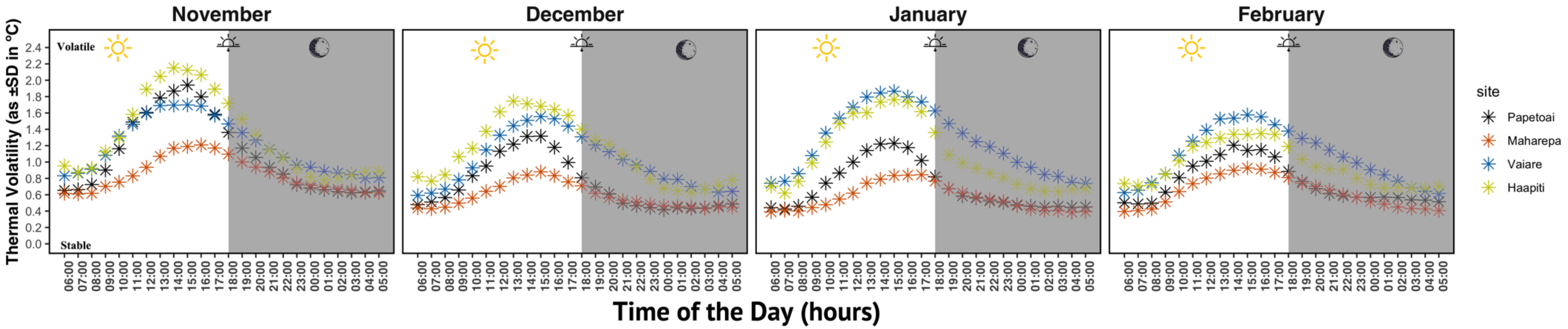

Figure S4. Thermal volatility (objective 5). Higher values (as SD) indicate thermal volatility, whereas lower values indicate thermal stability. Trends are given for the four parturition months: November through February, for each site
